# Association of body temperature and antipyretic treatments with mortality of critically ill patients with and without sepsis: multi-centered prospective observational study

**DOI:** 10.1186/cc11211

**Published:** 2012-02-28

**Authors:** Byung Ho Lee, Daisuke Inui, Gee Young Suh, Jae Yeol Kim, Jae Young Kwon, Jisook Park, Keiichi Tada, Keiji Tanaka, Kenichi Ietsugu, Kenji Uehara, Kentaro Dote, Kimitaka Tajimi, Kiyoshi Morita, Koichi Matsuo, Koji Hoshino, Koji Hosokawa, Kook Hyun Lee, Kyoung Min Lee, Makoto Takatori, Masaji Nishimura, Masamitsu Sanui, Masanori Ito, Moritoki Egi, Naofumi Honda, Naoko Okayama, Nobuaki Shime, Ryosuke Tsuruta, Satoshi Nogami, Seok-Hwa Yoon, Shigeki Fujitani, Shin Ok Koh, Shinhiro Takeda, Shinsuke Saito, Sung Jin Hong, Takeshi Yamamoto, Takeshi Yokoyama, Takuhiro Yamaguchi, Tomoki Nishiyama, Toshiko Igarashi, Yasuyuki Kakihana, Younsuck Koh

**Affiliations:** 1 Department of Anesthesiology, St. Paul's Hospital, Catholic University of Korea, Seoul, Republic of Korea; 2 Department of Emergency and Critical Care Medicine, Tokushima University Hospital, Tokushima, Japan; 3 Division of Pulmonary and Critical Care Medicine, Samsung Medical Center, Sungkyunkwan University School of Medicine, Seoul, Republic of Korea; 4 Department of Pulmonary and Critical Care Medicine, Chung-Ang University College of Medicine, Seoul, Republic of Korea; 5 Department of Anesthesiology and Pain Medicine, Pusan National University School of Medicine, Busan, Republic of Korea; 6 School of Media, Seoul Women's University, Seoul, Republic of Korea; 7 Department of Anesthesiology and Intensive Care Medicine, Hiroshima City Hospital, Hiroshima, Japan; 8 Division of Intensive and Coronary Care Unit, Nippon Medical School Hospital, Tokyo, Japan; 9 Tonami General Hospital, Toyama, Japan; 10 Intensive Care Division, Ehime University Hospital, Ehime, Japan; 11 Emergency & Critical Care Medicine, Akita University Graduate School of Medicine, Akita, Japan; 12 Department of Intensive Care, Okayama University Hospital, Okayama, Japan; 13 Department of Internal Medicine, Misato Kenwa Hospital, Saitama, Japan; 14 Intensive Care Unit, Department of Anesthesiology, Teine Keijinkai Hospital, Sapporo, Japan; 15 Department of Anesthesiology, Kyoto Prefectural University of Medicine, Kyoto, Japan; 16 Department of Anesthesiology and Pain Medicine, Seoul National University Hospital, Seoul, Republic of Korea; 17 Anesthesiology and Critical Cate Medicine, Konkuk University Hospital, Seoul, Republic of Korea; 18 Department of Anesthesiology and Critical Care Medicine, Saitama Medical Center, Jichi Medical University, Saitama, Japan; 19 Division of Intensive Care Medicine, Kagoshima University Hospital, Kagoshima, Japan; 20 Advanced Medical Emergency and Critical Care Center, Yamaguchi University Hospital, Yamaguchi, Japan; 21 Department of Anesthesiology and Pain Medicine, Chungnam National University Hospital, Daejeon, Republic of Korea; 22 Department of Emergency and Critical Care Medicine, St. Marianna University, Kanagawa, Japan; 23 Department of Anesthesiology and Pain Medicine, Anesthesia and Pain Research Institute, Severance Hospital, Yonsei University College of Medicine, Seoul, Republic of Korea; 24 Department of Anesthesiology and Pain Medicine, Incheon St Mary's Hospital, Catholic University of Korea, Medical College, Incheon, Republic of Korea; 25 Innovation of New Biomedical Engineering Center, Tohoku University, Sendai, Japan; 26 Department of Anesthesiology and Critical Care, Kamagaya General Hospital, Kamagaya, Japan; 27 Division of Pulmonary and Critical Care Medicine, Department of Internal Medicine, Asan Medical Center, University of Ulsan College of Medicine, Seoul, republic of Korea

**Keywords:** body temperature, antipyretic, fever, critical illness, mortality

## Abstract

**Introduction:**

Fever is frequently observed in critically ill patients. An independent association of fever with increased mortality has been observed in non-neurological critically ill patients with mixed febrile etiology. The association of fever and antipyretics with mortality, however, may be different between infective and non-infective illness.

**Methods:**

We designed a prospective observational study to investigate the independent association of fever and the use of antipyretic treatments with mortality in critically ill patients with and without sepsis. We included 1,425 consecutive adult critically ill patients (without neurological injury) requiring > 48 hours intensive care admitted in 25 ICUs. We recorded four-hourly body temperature and all antipyretic treatments until ICU discharge or 28 days after ICU admission, whichever occurred first. For septic and non-septic patients, we separately assessed the association of maximum body temperature during ICU stay (MAX_ICU_) and the use of antipyretic treatments with 28-day mortality.

**Results:**

We recorded body temperature 63,441 times. Antipyretic treatment was given 4,863 times to 737 patients (51.7%). We found that treatment with non-steroidal anti-inflammatory drugs (NSAIDs) or acetaminophen independently increased 28-day mortality for septic patients (adjusted odds ratio: NSAIDs: 2.61, *P *= 0.028, acetaminophen: 2.05, *P *= 0.01), but not for non-septic patients (adjusted odds ratio: NSAIDs: 0.22, *P *= 0.15, acetaminophen: 0.58, *P *= 0.63). Application of physical cooling did not associate with mortality in either group. Relative to the reference range (MAX_ICU _36.5°C to 37.4°C), MAX_ICU _≥ 39.5°C increased risk of 28-day mortality in septic patients (adjusted odds ratio 8.14, *P *= 0.01), but not in non-septic patients (adjusted odds ratio 0.47, *P *= 0.11).

**Conclusions:**

In non-septic patients, high fever (≥ 39.5°C) independently associated with mortality, without association of administration of NSAIDs or acetaminophen with mortality. In contrast, in septic patients, administration of NSAIDs or acetaminophen independently associated with 28-day mortality, without association of fever with mortality. These findings suggest that fever and antipyretics may have different biological or clinical or both implications for patients with and without sepsis.

**Trial registration:**

ClinicalTrials.gov: NCT00940654

## Introduction

Fever frequently occurs in critically ill patients [1]. Although fever is primarily a symptom of infection [2], it also occurs as a host's response to non-infectious inflammatory stimulus [3]. Currently, the effect of antipyretics on patient outcomes remains unclear and there are no recommendations for antipyretic treatments for non-neurological critically ill patients [2,4].

Fever may have detrimental effects, especially in life-threatening illnesses, by increasing the metabolic rate, minute ventilation and oxygen consumption, and by adversely affecting neurological outcomes [5-7]. Thus, antipyretic treatments are frequently administered in critically ill patients both with and without infectious diseases [8-10].

Fever below a fatal temperature, however, could be a host response against infectious disease resulting in reduced bacterial growth, promotion of the synthesis of antibodies and cytokines, and activation of T cells, neutrophils and macrophages [11-13]. Several studies have suggested that suppression of infective febrile responses with antipyretic treatments might worsen outcomes [14,15].

Based on the studies mentioned above, it would be desirable to understand whether there is an independent association of fever and the use of antipyretic treatments with mortality in infective critical ill patients and whether this association is the same in non-infective critical ill patients.

Accordingly, we conducted a multicenter prospective observational study to test the hypothesis that an independent association of fever and antipyretic treatments with mortality was significantly modified by the presence of sepsis at admission to the ICU.

## Materials and methods

### Study design

This study was a prospective observational investigation conducted in 25 hospitals: 10 in Korea and 15 in Japan. Among these 25 hospitals, 20 were academic tertiary care hospitals and 5 were community hospitals. Participating hospitals range in size from 248 to 2,860 beds (median of 736) and included a total of 1,002 ICU beds (median of 20 beds per ICU). Data collection and data analysis for this study were approved by each of the local institutional ethics committees, and each waived the requirement for informed consent.

### Patients

At each participating site, all adult patients who required intensive care for more than 48 hours from 1 September 2009 to 30 November 2009 were candidates for enrollment in the study; we excluded patients with post-cardiac arrest, post craniotomy, traumatic brain injury, central nervous system infection, subarachnoid hemorrhage, intracerebral hemorrhage or stroke at their ICU admission.

We separated our cohorts into patients with and without sepsis for the first 24 hours of ICU admission. Sepsis was defined as the presence of microbiologically proven, clinically affirmed or suspected infection along with the presence of systemic inflammatory response syndrome [16,17].

### Data collection

#### Demographic data

Age, sex, reason for admission, use of mechanical ventilation and Acute Physiology and Chronic Health Evaluation (APACHE) II score [18] were recorded. Coding for major admission diagnosis was categorized as cardiac or vascular disease, thoracic or respiratory disease, renal or metabolic disease, gastrointestinal tract disease, and other.

#### Body temperature

We recorded four-hourly body temperature until either time of ICU discharge or 28 days after ICU admission, whichever occurred first. Measuring methods included use of pulmonary artery and bladder catheter thermistors, tympanic membrane and axillary thermometers. When body temperature was simultaneously measured using more than one method, we recorded the value measured by the method most preferred by the American College of Critical Care Medicine and the Infectious Diseases Society of America [2].

#### Information for antipyretic treatments

While no standardized protocols for the prevention or treatment of fever were applied across the participating ICUs, we recorded all the antipyretic treatments during ICU stay; including non-steroidal anti-inflammatory drugs (NSAIDs), acetaminophen and physical cooling. Body temperature at commencement of antipyretic treatment was also recorded. Information for NSAIDs and acetaminophen was recorded only when these were administered for fever management, not for pain control. Physical cooling methods included external air and water blanket techniques, and internal cold gastric lavage or cold fluid infusion.

### Outcome

The primary outcome of interest was mortality up to 28 days after ICU admission, and the association of this with peak body temperature during ICU stay and administered antipyretic treatments. Patients who were discharged alive from the hospital before Day 28 were defined as survived.

### Statistical analysis

Categorical variables were summarized using proportions and compared between groups using the chi-square test and continuous variables were summarized using mean (SD) or median (interquartile range; IQR) and compared between groups using Student's *t*-test or the Wilcoxon rank-sum test as appropriate.

To determine the severity of fever, we considered maximum body temperature (MAX_ICU_), the highest body temperature recorded during ICU stay. As the relationship between body temperature and mortality may not be linear, we treated body temperature as a categorical variable. MAX_ICU _was analyzed in five range categories: (A) < 36.5°C, (B) 36.5°C to 37.4°C, (C) 37.5°C to 38.4°C, (D) 38.5°C to 39.4°C and (E) ≥ 39.5°C. Odds ratios are reported relative to a reference body temperature, defined here as category (B) (36.5°C to 37.4°C). Additionally, we performed survival log-rank test to compare each range categories.

We performed multivariate logistic regression analysis, treating as independent variables, site, age, use of mechanical ventilation, APACHE-II score with the body temperature component removed [18], category of ICU admission, whether surgical or medical admission, subgroup of MAX_ICU_, application of antipyretic treatment; the dependent variable was death within 28 days of admission. Model calibration was determined using the Hosmer-Lemeshow test for goodness of fit. Results from the multivariate models are reported using odds ratios with 95% confidence intervals.

For sensitivity analysis, we further developed another multivariate model among patients with lowest body temperature during ICU stay > 35°C. We defined this threshold (> 35°C) as a recent large epidemiological study defined 35°C as the threshold of moderate to severe hypothermia [19].

We assumed 50% of the study patients had sepsis, a quarter of them were prescribed antipyretic therapy and the mortality of ICU patients was 12%. Assuming an 8% change in ICU mortality with antipyretic both in septic and non-septic patients, a power of 0.80, and an α level of 0.05, we required 1,400 participants. As we expected to include approximately 500 participants per month, we planned to conduct the current study for three months.

*P-*values of less than 0.05 were considered statistically significant. All analyses were performed using commercially available statistical software (SPSS 19.0, SPSS Inc, Chicago, IL, USA). Data are reported in accordance with the guidelines laid out in Strengthening the Reporting of Observational Studies in Epidemiology (STROBE) [20].

## Results

**Figure 1:**
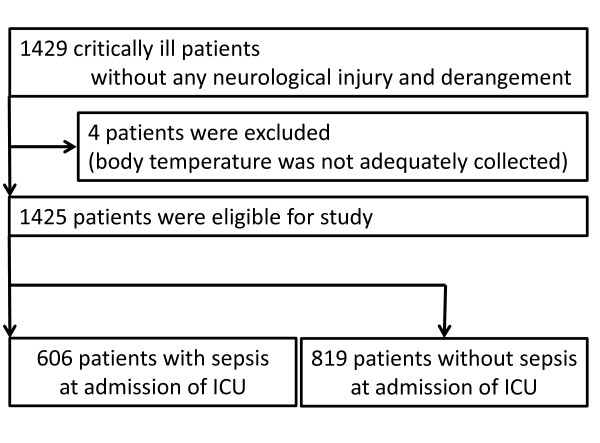
**Flow chart showing current study**. ICU, intensive care units.

We studied 1,429 consecutive patients. We excluded four patients for whom data were incomplete (4/1,429, 0.3%), leaving a total of 1,425 patients with 63,441 body temperature measurements eligible for inclusion in the study. Seventy-two percent of body temperature was measured by axillary thermometers, 16% by bladder catheter thermistors, 9% by tympanic membrane thermometers and 3% by pulmonary artery catheter thermistors. The median APACHE II score was 17, and 28-day mortality was 12.0%. The median length of stay in ICU was 7 days and in hospital 26 days. Among the 1,425 patients, 606 patients met the criteria for sepsis during the first 24 h. The remaining 819 patients were without sepsis (Figure 1).

Table 1 provides clinical characteristics, MAX_ICU _and antipyretic treatments for septic and non-septic patients and for the total cohort. Septic patients tended to be more severely ill, older and were less likely to be post-operative patients or to have required mechanical ventilation during ICU stay. The 28-day mortality in septic patients was significantly higher than in non-septic patients.

**Table 1:** Comparison of baseline characteristics of patients with and without sepsis

	Patients with sepsis (*N *= 606)	Patients without sepsis (*N *= 819)	*P*-value	Total cohort (*N *= 1425)
28-day mortality n (%)	135 (22.3%)	36 (4.4%)	< 0.001	171 (12.0%)
Gender (male) n (%)	385 (63.5%)	508 (62%)	0.56	893 (62.7%)
Age (y.o, IQR)	67 (55, 75)	65 (54, 73)	0.015	66 (54, 74)
APACHE II score (IQR)	21 (16, 25)	14 (10, 18)	< 0.001	17 (12, 22)
Mechanical ventilation requirement n (%)	429 (70.8%)	528 (64.5%)	0.01	957 (67.2%)
Postoperative admission n (%)	68 (11.2%)	538 (65.7%)	< 0.001	602 (42.2%)
Reasons for admission n (%)				
Cardiac or vascular disease	109 (18.0%)	435 (53.1%)	< 0.001	544 (38.2%)
Thoracic or respiratory disease	343 (56.6%)	205 (25.0%)	< 0.001	548 (38.4%)
Renal or metabolic disease	61 (10.0%)	54 (6.6%)	0.018	115 (8.1%)
Gastrointestinal tract disease	68 (11.2%)	108 (13.1%)	0.26	176 (12.4%)
Other	25 (4.1%)	17 (2.1%)	0.024	42 (2.9%)
Length of stay in ICU	8 (5, 14)	5 (4, 7)	< 0.001	6 (4, 10)
**MAX_ICU _**(°C, IQR)	38.3 (37.7, 39.0)	37.8 (37.4, 38.3)	< 0.001	38.0 (37.5, 38.6)
< 36.5°C	4 (0.7%)	2 (0.2%)	0.43	6 (0.4%)
36.5°C to 37.4°C	98 (16.2%)	240 (29.3%)	< 0.001	338 (23.7%)
37.5°C to 38.4°C	237 (39.1%)	425 (52.0%)	< 0.001	662 (46.5%)
38.5°C to 39.4°C	185 (30.5%)	125 (15.3%)	< 0.001	310 (21.8%)
≥ 39.5°C	82 (13.5%)	27 (3.3%)	< 0.001	109 (7.6%)
**NSAIDs**				
Number of patients administered n (%)	31 (5.1%)	99 (12.1%)	< 0.001	130 (9.1%)
Delta body temperature* (°C, IQR)	-0.3 (-0.8, 0.0)	-0.4 (-0.7, 0.0)	0.57	-0.3 (-0.7, 0.0)
**Acetaminophen**				
Number of patients administered n (%)	116 (19.1%)	32 (3.9%)	< 0.001	148 (10.4%)
Delta body temperature* (°C, IQR)	-0.4 (-0.8, -0.2)	-0.3 (-0.7, 0.0)	0.14	-0.4 (-0.9, -0.2)
**Physical cooling**				
Number of patients administered n (%)	307 (50.7%)	364 (44.4%)	0.02	671 (47.1%)
Delta body temperature* (°C, IQR)	-0.2 (-0.5, 0.0)	-0.1 (-0.3, 0.0)	< 0.001	-0.1 (-0.3, 0.0)

APACHE, Acute Physiology and Chronic Health Evaluation; IQR, 25%, 75% interquartile; MAX_ICU_, maximum body temperature during ICU stay; NSAIDs, non-steroid anti-inflammatory drugs

*, Delta body temperature from application of antipyretic to next temperature monitoring

**Figure 2:**
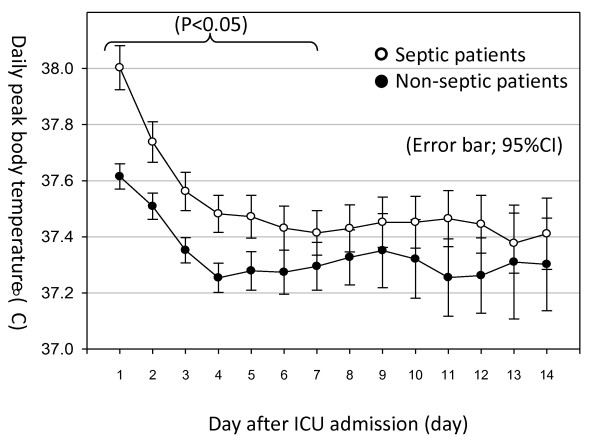
**Mean peak daily temperature of patients with and without sepsis**. The white circles indicate the mean peak daily temperature in patients with sepsis. The black circles indicate the mean peak daily temperature in patients without sepsis. For the first seven days after admission, peak body temperature of patients with sepsis was significantly higher than of patients without sepsis. CI, confidential interval; ICU, intensive care unit.

Septic patients exhibited significantly higher MAX_ICU _than non-septic patients (38.3°C vs. 37.8°C, *P *< 0.001). MAX_ICU _in septic patients was more frequently in the higher range (≥ 38.5°C) (Table 1). This difference was present for the first seven days of ICU stay (Figure 2).

Table 2 shows 28-day mortality and odds ratio for each range of MAX_ICU _relative to the reference range of 36.5°C to 37.4°C in septic and non-septic patients, respectively. Mortality did not relate to MAX_ICU _in septic patients. By contrast, for non-septic patients, 28-day mortality increased according to MAX_ICU_, and was significantly greater when MAX_ICU _was ≥ 38.5°C (odds ratio; 5.13 (*P *< 0.007) and 13.4 (*P *< 0.001), 38.5°C to 39.4°C and ≥ 39.5°C, respectively). There was no significant difference in 28-day mortality between patients with single and multiple episodes of MAX_ICU _≥ 39.5°C. Mortality for septic patients with MAX_ICU _< 36.5°C was as high as 50%, but no significant difference was detected owing to the small number of patients in this category (*n *= 4) (odds 3.08, *P *= 0.57).

**Table 2:** Maximum body temperature during ICU stay and 28-day mortality of patients with and without sepsis.

**MAX**_**ICU**_	Patients with sepsis (*N *= 606)		Patients without sepsis (*N *= 819)	
	
	28-day mortality	Unadjusted odds ratio (95% CI)	28-day mortality	Unadjusted odds ratio (95% CI)
< 36.5°C	2/4 (50.0%)	3.08 (0.41, 23.1) (*P *= 0.57)	0/2 (0%)	n.a.
36.5°C to 37.4°C	24/98 (24.5%)	1 (reference)	4/240 (1.7%)	1 (reference)
37.5°C to 38.4°C	40/237 (16.9%)	0.63 (0.35, 1.11) (*P *= 0.14)	17/425 (4.0%)	2.46 (0.82, 7.40) (*P *= 0.44)
38.5°C to 39.4°C	44/185 (23.8%)	0.96 (0.54, 1.70) (*P *= 0.99)	10/125 (8.0%)	5.13 (1.58, 16.7) (*P *= 0.007)
≥ 39.5°C	25/82 (30.5%)	1.35 (0.70, 2.61) (*P *= 0.46)	5/27 (18.5%)	13.4 (3.35, 53.6) (*P *< 0.001)

Unadjusted odds ratio was reported relative to a reference body temperature defined as category 36.5°C to 37.4°C.

CI, confidential interval; ICU, intensive care unit; MAX_ICU_, maximum body temperature during an ICU stay; n.a., not applicable (no patients in this category)

**Figure 3:**
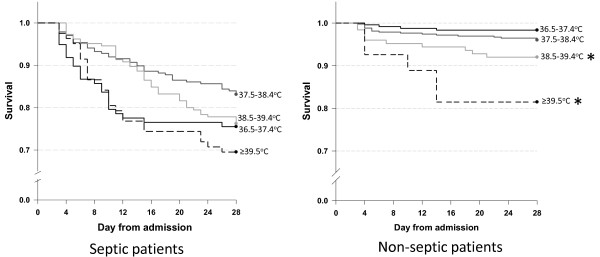
**Maximum body temperature during ICU stay and survival of patients with and without sepsis**. This figure shows Kaplan-Meier estimates for the probability of survival, which at 28 days was greater in non-septic patients with MAX_ICU _38.5°C to 39.4°C and ≥ 39.5°C than those with 36.5°C to 37.4°C. In septic patients, there were no significant differences of provability of survival in each category compared with patients of MAX_ICU _with 36.5°C to 37.4°C. *, significantly different probability of survival at 28 days after ICU admission than patients with 36.5°C to 37.4°C.

Figure 3 shows Kaplan-Meier estimates for the probability, which at 28 days was greater in non-septic patients with MAX_ICU _≥ 38.5°C than those with temperatures of 36.5°C to 37.4°C. In septic patients, there were no significant differences of provability of survival in each category compared with patients of MAX_ICU _with 36.5°C to 37.4°C.

Antipyretic treatment was applied 4,863 times to 737 patients (51.7%). NSAIDs were administered 429 times to 130 patients (9.1%), acetaminophen was administered 571 times to 148 patients (10.4%) and physical cooling was applied 3,863 times to 671 patients (47.1%). Delta body temperature from application of antipyretic to next temperature monitoring was -0.3°C (IQR; -0.7, 0.0) and -0.4°C (IQR; -0.9, -0.2), for NSAIDs and acetaminophen respectively, which is significantly greater than -0.1°C for physical cooling (*P *< 0.001) (Table 1).

**Figure 4:**
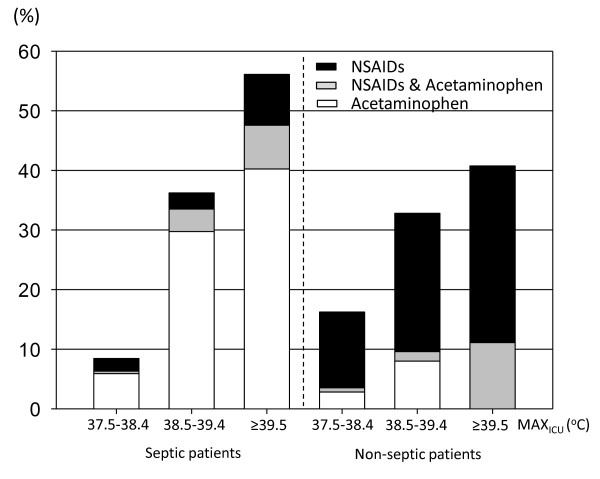
**Administration of pharmacological antipyretic treatments (NSAIDs and/or acetaminophen) in each MAX_ICU _category**. Data show patients categorized in subgroups according to MAX_ICU _value range: 37.5°C to 38.4°C, 38.5°C to 39.4°C and ≥ 39.5°C. White bar, patients given NSAIDs; black bar, patients given acetaminophen; gray bar, patients given both NSAIDs and acetaminophen. For the subgroup with MAX_ICU _of 37.5°C to 38.4°C, the proportion of patients received pharmacological antipyretic treatments was significantly higher in non-septic patients (*P *= 0.007). For the rest of the subgroups, it was not significantly different between patients with and without sepsis (38.5°C to 39.4°C, *P *= 0.62; ≥ 39.5°C, *P *= 0.25). Acetaminophen was used more frequently for patients with sepsis, and NSAIDs for patients without sepsis in each MAX_CAT _subgroup (*P *< 0.001). MAX_ICU_, maximum body temperature recorded during ICU stay; NSAIDs: non-steroid anti-inflammatory drugs.

**Figure 5:**
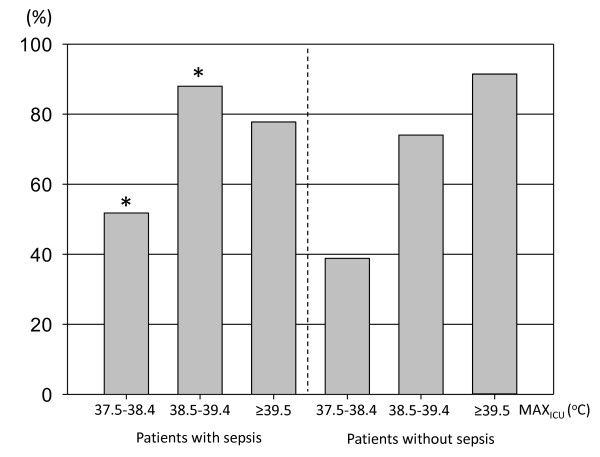
**Use of physical cooling in each MAX_ICU _category of patients with and without sepsis**. Data show patients categorized in subgroups according to MAX_ICU _value range: 37.5°C to 38.4°C, 38.5°C to 39.4°C, and ≥ 39.5°C. *statistically significant difference between patients with and without sepsis. MAX_ICU_, maximum body temperature recorded during ICU stay.

Figure 3 shows the proportion of patients who received pharmacological antipyretic treatments (NSAIDs, acetaminophen or both) in each subgroup. For the subgroup with MAX_ICU _of 37.5°C to 38.4°C, the proportion of patients who received pharmacological antipyretic treatments was significantly higher than in non-septic patients (*P *= 0.007). For the rest of the subgroups, it was not significantly different between patients with and without sepsis (38.5°C to 39.4°C, *P *= 0.62; ≥ 39.5°C, *P *= 0.25). Acetaminophen was used more frequently for septic patients, and NSAIDs for non-septic patients in each range of MAX_ICU _(*P *< 0.001) (Figure 3). Figure 4 shows the proportion of septic and non-septic patients who received physical cooling. Physical cooling was applied frequently in septic patients, when MAX_ICU _was ≤ 39.4°C (Figure 5).

Table 3 shows univariate comparisons of patient demographic data and antipyretic treatments. In septic patients, the use of NSAIDs and acetaminophen was significantly associated with increased mortality (odds ratio: NSAIDs 2.32, *P *= 0.02; acetaminophen 2.30, *P *= 0.002) (Table 3). By contrast, for non-septic patients, the pharmacological antipyretic treatments were not associated with mortality (odds ratio: NSAIDs 0.20, *P *= 0.08; acetaminophen 0.69, *P *= 0.72). Physical cooling was associated with mortality in neither septic nor non-septic patients (odds ratio: with sepsis 1.00, *P *= 0.99; without sepsis 1.14, *P *= 0.74).

**Table 3:** Comparison of baseline characteristics and antipyretic treatments of survivors and non-survivors

	Patients with sepsis			Patients without sepsis		
	**Survivors** **(*N *= 471)**	**Non-survivors** **(*N *= 135)**	***P*-value**	**Survivors** **(*N *= 783)**	**Non-survivors** **(*N *= 36)**	***P*-value**

Gender (male) n (%)	291 (61.8%)	94 (69.6%)	0.10	485 (61.9%)	23 (63.9%)	0.81
Age (y.o., IQR)	67 (54, 75)	69 (58, 78)	0.08	64 (54, 73)	74.5 (59, 81.5)	0.003
APACHE (IQR)	20 (16, 24)	24 (21, 28)	< 0.001	13 (10, 18)	24 (19.5, 28)	< 0.001
Postoperative admission n (%)	59 (12.5%)	9 (6.7%)	0.057	523 (66.8%)	11 (30.6%)	< 0.001
Mechanical ventilation requirement n (%)	317 (67.3%)	112 (83.0%)	< 0.001	498 (63.6%)	30 (83.3%)	0.016
Reasons for admission n (%)						
Cardiac or vascular diseases	93 (19.7%)	16 (11.9%)	0.04	422 (53.9%)	13 (36.1%)	0.04
Thoracic or respiratory diseases	253 (53.7%)	90 (66.7%)	0.007	193 (24.6%)	12 (33.3%)	0.24
Renal or metabolic disease	48 (10.2%)	13 (9.6%)	0.85	51 (6.5%)	3 (8.3%)	0.67
Gastrointestinal tract diseases	56 (11.9%)	12 (8.9%)	0.33	102 (13%)	6 (16.7%)	0.53
Other	21 (4.5%)	4 (3%)	0.44	15 (1.9%)	2 (5.6%)	0.13
Length of stay in ICU	8 (5, 14)	9 (5, 15)	0.74	5 (4, 7)	5 (4, 9)	0.11

***NSAIDs***						
Number of patients n (%) (Un adjusted odds ratio (95% CI))	19 (4%)	12 (8.9%)	0.02	98 (12.5%)	1 (2.8%)	0.08
	(2.32 (1.10, 4.91))			(0.20 (0.03, 1.47))		
Delta body temperature* (°C, IQR)	-0.4 (-0.9, -0.1)	-0.1 (-0.6, 0.0)	0.08	-0.4 (-0.7, 0)	0.0 (0.0, 0.0)	0.30

**Acetaminophen n (%)**						
Number of patients n (%) (Un adjusted odds ratio (95%CI))	75 (15.9%)	41 (30.4%)	0.002	31 (4%)	1 (2.8%)	0.72
	(2.30 (1.48, 3.58))			(0.69 (0.09, 5.23))		
Delta body temperature* (°C, IQR)	-0.4 (-1.1, -0.1)	-0.4 (-0.9, -0.2)	0.94	-0.3 (-0.7, 0.0)	-0.9 (-0.9, -0.9)	0.28

**Cooling n (%)**						
Number of patients n (%) (Unadjusted odds ratio (95%CI))	223 (47.3%)	64 (47.4%)	0.99	366 (46.7%)	18 (50%)	0.74
	(1.00 (0.68, 1.46))			(1.14 (0.58, 2.22))		
Delta body temperature* (°C, IQR)	-0.2 (-0.4, 0)	-0.2 (-0.5, 0)	0.55	-0.1 (-0.3, 0)	-0.1 (-0.4, 0)	0.16

APACHE, Acute Physiology and Chronic Health Evaluation; CI, confidential interval; IQR, 25%, 75% interquartile; NSAIDs, non-steroid anti-inflammatory drugs

*, Delta body temperature from application of antipyretic to next temperature monitoring

### Multivariate analysis

As shown in Table 3, the presence of confounders, such as severity of illness, age, reason for ICU admission and mechanical ventilation requirement, necessitated multivariate analysis adjusting for relevant predictors of 28-day mortality.

In septic patients relative to the reference range (36.5°C to 37.4°C), MAX_ICU _37.5°C to 38.4°C was associated with decreased mortality (adjusted odds ratio 0.45, *P *= 0.014) and MAX_ICU _≥ 38.5°C was not (adjusted odds ratio 38.5°C to 39.4°C; 0.52, *P *= 0.09, ≥ 39.5°C; 0.47, *P *= 0.11). In non-septic patients, adjusted risk of death was increased as MAX_ICU _increased, and MAX_ICU _≥ 39.5°C was associated with mortality (adjusted odds ratio 8.14, *P *= 0.01) (Table 4).

**Table 4:** Multivariate logistic analysis for 28-day mortality

	Patients with sepsis (*N *= 606)		Patients without sepsis (*N *= 819)	
	
	Adjusted odds ratio (95% CI)	*P*-value	Adjusted odds ratio (95% CI)	*P*-value
Age (y.o., IQR)	1.01 (0.99, 1.02)	0.32	1.01 (0.98, 1.04)	0.39
APACHE (IQR)	1.09 (1.05, 1.12)	< 0.001	1.19 (1.12, 1.27)	< 0.001
Postoperative admission n (%)	0.81 (0.36, 1.85)	0.62	0.56 (0.24, 1.29)	0.17
Mechanical ventilation requirement n (%)	1.72 (1.01, 2.94)	0.045	3.85 (1.40, 10.6)	0.01
Cardiac or vascular diseases	0.87 (0.43, 1.76)	0.70	1.03 (0.38, 2.75)	0.96
Thoracic or respiratory diseases	1.71 (0.99, 2.94)	0.053	1.87 (0.70, 4.97)	0.21

Max. body temp. during ICU stay				
37.5°C to 38.4°C (vs. 36.5°C to 37.4°C)	0.45 (0.24, 0.85)	0.014	1.61 (0.56, 4.68)	0.38
38.5°C to 39.4°C (vs. 36.5°C to 37.4°C)	0.52 (0.24, 1.1)	0.09	3.34 (0.88, 12.69)	0.08
≥ 39.5°C (vs. 36.5°C -37.4°C)	0.47 (0.19, 1.18)	0.11	8.14 (1.67, 39.59)	0.01

NSAIDs	2.61 (1.11, 6.11)	0.028	0.22 (0.03, 1.74)	0.15
Acetaminophen	2.05 (1.19, 3.55)	0.01	0.58 (0.06, 5.26)	0.63
Cooling	1.2 (0.70, 2.05)	0.50	0.71 (0.30, 1.69)	0.44

APACHE, Acute Physiology and Chronic Health Evaluation; CI, confidential interval; MAX_ICU_, maximum body temperature during an ICU stay; NSAIDs, non-steroidal anti-inflammatory drugs

Subgroup with MAX_ICU _< 36.5°C was excluded from this analysis because only three patients with sepsis and none without fell within this temperature range.

In septic patients, administration of NSAIDs or acetaminophen was independently associated with mortality (adjusted odds ratio: NSAIDs 2.61, *P *= 0.028; acetaminophen 2.05, *P *= 0.01). In non-septic patients, the pharmacological antipyretic treatments were not associated with mortality (adjusted odds ratio: NSAIDs 0.22, *P *= 0.15; acetaminophen 0.58, *P *= 0.63) (Table 4). The model was a good fit for data from both groups (Hosmer-Lemeshow: with sepsis, *P *= 0.21, without sepsis, *P *= 0.89).

To exclude the possible effects of hypothermia, we further performed multivariate logistic analysis excluding data from 106 patients with low body temperature (≤ 35°C) during ICU stay (Table 5). Even in this model, administration of NSAIDs and acetaminophen in septic patients was independently associated with mortality (adjusted odds ratio: NSAIDs 2.48, *P *= 0.04; acetaminophen 1.95, *P *= 0.03). Meanwhile, for non-septic patients, MAX_ICU _≥ 38.5°C was associated with mortality (adjusted odds ratio 38.5°C to 39.4°C; 7.49, *P *= 0.02, ≥ 39.5°C; 11.7, *P *= 0.02).

**Table 5:** Multivariate logistic analysis for 28-day mortality in patients with lowest body temperature > 35°C

	Patients with sepsis (*N *= 507)		Patients without sepsis (*N *= 715)	
	
	Adjusted odds ratio (95% CI)	*P*-value	Adjusted odds ratio (95% CI)	*P*-value
Age (y.o., IQR)	1.02 (1.00, 1.03)	0.08	1.01 (0.98, 1.05)	0.52
APACHE (IQR)	1.07 (1.03, 1.11)	< 0.001	1.20 (1.11, 1.30)	< 0.001
Postoperative admission n (%)	0.79 (0.32, 1.91)	0.59	0.57 (0.21, 1.52)	0.26
Mechanical ventilation requirement n (%)	1.76 (0.98, 3.14)	0.06	3.49 (1.14, 10.7)	0.03
Cardiac or vascular diseases	0.85 (0.39, 1.82)	0.67	0.73 (0.24, 2.21)	0.57
Thoracic or respiratory diseases	1.23 (0.66, 2.28)	0.51	1.24 (0.40, 3.78)	0.71

**MAX**_**ICU**_				
37.5°C to 38.4°C (vs. 36.5°C to 37.4°C)	0.42 (0.20, 0.88)	0.02	3.18 (0.78, 12.9)	0.11
38.5°C to 39.4°C (vs. 36.5°C to 37.4°C)	0.60 (0.25, 1.43)	0.25	7.49 (1.47, 38.1)	0.02
≥ 39.5°C (vs. 36.5°C to 37.4°C)	0.59 (0.21, 1.68)	0.32	11.7 (1.58, 87.0)	0.02

**NSAIDs**	2.48 (1.02, 6.01)	0.04	0.26 (0.032, 2.19)	0.22
**Acetaminophen**	1.95 (1.06, 3.57)	0.03	0.61 (0.23, 1.61)	0.32
**Cooling**	1.23 (0.69, 2.21)	0.47	1.26 (0.14, 11.0)	0.84

APACHE, Acute Physiology and Chronic Health Evaluation; CI, confidential interval; MAX_ICU_, maximum body temperature during an ICU stay; NSAIDs, non-steroid anti-inflammatory drugs

## Discussion

### Key results

We performed a study to explore the relationship of fever and antipyretic treatments with 28-day mortality in critically ill patients and to quantify the difference of its association between patients with and without sepsis. We found that in septic patients, compared with the 36.5°C to 37.4°C subgroup, MAX_ICU _37.5°C to 38.4°C was associated with decreased mortality and MAX_ICU _≥ 38.5°C was not independently associated with mortality. By contrast, in non-septic patients, high fever (≥ 39.5°C) was independently associated with mortality. We also found significant interactions between mortality and treatment with NSAIDs or acetaminophen only in septic patients.

### Limitations of this study

Our study had several limitations. First, because it was designed as an observational study without standardized protocols for antipyretic treatments, the findings can only show association and not causality. Thus, our results can only be viewed as useful for generating hypotheses.

The methods of body temperature monitoring were not standardized. Furthermore, the majority of the body temperatures was measured by axillary thermometers, although core temperature is less influenced by external factors and more accurately reflects temperature of the vital organs [21]. Additionally, it is possible that the sickest patients were more likely to have had invasive measurements of core temperature, resulting in relatively higher values. The proportion of methods of body temperature monitoring, however, used in septic patients was not significantly different from non-septic patients. Thus, any bias-related body temperature monitoring would similarly influence both cohorts. Nonetheless, our finding may be accentuated due to changes in circulation occurring during the progression of sepsis environmental temperature [22]. In this regard, our finding should be confirmed or refuted by further studies using core body temperature monitoring.

Although the proportion of patients treated with pharmacological antipyretic treatments were similar in both septic and non-septic patients, NSAIDs were more frequently administered to non-septic patients, while acetaminophen was used more frequently for septic patients. Additionally, acetaminophen was less frequently administered in the present study than in other studies. Young *et al*. reported that acetaminophen was administered to 58% to 70% of septic patients [10], while only to about 20% in our patients. Physical cooling was applied more frequently in our population than reported elsewhere [10]. These facts may influence their association with mortality. Thus, we duly note that our findings may not be applicable to other settings where antipyretic procedures are different. Additionally, we studied in only two countries and our findings may not be generalizable to other countries, especially those with different medical systems.

We used delta body temperature after antipyretic treatments to compare the strength of each antipyretic effect. We should note that this index did not reflect the absolute reduction of body temperature after antipyretic procedures, as the timing of temperature measurement after antipyretics was not standardized and there might not be a linear relationship between individual antipyretic treatment and the next body temperature.

Finally, we excluded critically ill patients with neurological injury. It is widely accepted that fever adversely affects the outcome of critically ill patients with neurological injury [23,24] and should be treated [6,7]. By contrast, there is no clear recommendation for antipyretic treatments for non-neurological critically ill patients [2,4,25]. Thus, we chose to exclude patients with neurological injury.

### Interpretation

Although we reported associations and cannot assume causality, our finding was consistent with previous studies [26,27], suggesting that fever could be a host response that protects against infectious diseases [11-13] and use of antipyretic treatments to suppress the febrile response to infection might worsen outcomes [14,15].

The association of fever with mortality varied according to the level of fever and it was independently associated with mortality only in subgroup ≥ 39.5°C of patients without sepsis. We assume that high fever seen in patients without sepsis was likely caused by infection subsequent to ICU admission and this may account for the higher mortality. We did find, however, that the association of MAX_ICU _and mortality were similar in patients with no sign of infection during ICU stay (Additional file 1).

A large epidemiological study has revealed that the presence of fever (≥ 38.3°C) is not associated with increased ICU mortality (13% vs. 12%: *P *= 0.08), but that high fever (≥ 39.5°C) was associated with significantly increased mortality (20.3% vs. 12%, *P *< 0.001) [1]. High fever can result in cardiac arrhythmias, tachycardia, increased oxygen demand, convulsions and brain damage [5-7]. Patients with non-infective febrile responses may experience these deleterious effects without the potential benefit of fever-related protection against viruses or bacteria or both.

We found that the association with mortality of the administration of NSAIDs or acetaminophen or both was significantly different for non-septic patients than for septic patients. There are at least four possible explanations for our findings related to septic patients. First, the infective febrile response may be effective and lowering body temperature with antipyretics might be undesirable for septic patients. Fever is thought to inhibit the activity of viruses and bacteria [11-13] and antipyretic treatments shown to worsen outcomes in various animal and human studies [14,15]. The apparent antipyretic effects of NSAIDs and acetaminophen compared with physical cooling seen in our study lends weight to this hypothesis.

Second, in septic patients, administration of NSAIDs and acetaminophen may be toxic, as they might be associated with hypotension and renal dysfunction [28,29]. We could not assess this hypothesis in the current study.

Third, mortality is higher for septic patients who fail to develop a fever. We found the unadjusted mortality was as high as 66% in patients with MAX_ICU _< 36.5°C. This finding may also support the argument that fever is naturally protective. For sensitive analysis to avoid the bias of hypothermia (defined as lowest body temperature < 35.0°C.), we performed further multivariate analysis and found a similar association of the use of pharmacological antipyretic with mortality. Fourth, any combination of the above factors might also apply.

There is limited information on the effect of antipyretics on patient outcomes and there are no recommendations for antipyretic treatments for febrile patients with or without infectious diseases [2,4]. One study of trauma patients was abandoned early with the 82^nd ^patient on instruction of a safety monitoring board. This study reported the trend toward increased risk of infection and death in patients when acetaminophen and physical cooling were aggressively used [30]. Additionally, two studies reported that short-term therapy with ibuprofen in patients with sepsis did not influence mortality [31,32]. To our knowledge, this study is the first multicenter examination of the epidemiology and outcome associations of antipyretic treatments [33]. While the clinical benefits and risk of antipyretic treatments can only be properly assessed in a randomized controlled trial; until such time, our findings do have some practical implications.

## Conclusions

In conclusion, the association with mortality of fever and type of antipyretic treatment was different between patients with and without sepsis at admission to ICU. For non-septic patients, MAX_ICU _≥ 39.5°C was associated with 28-day mortality. Meanwhile, for septic patients, administration of NSAIDs and acetaminophen was independently associated with increased mortality. Since many ICU patients are or become febrile and antipyretic treatments are common, further studies now appear desirable to confirm or refute our observations.

## Key messages

• The association of fever with mortality was different for patients with and without sepsis at admission to ICU.

• For patients without sepsis, MAX_ICU _≥ 39.5°C was associated with 28-day mortality.

• The association of pharmacological antipyretic treatments with mortality was different for patients with and without sepsis at admission of ICU.

• For patients without sepsis, administration of NSAIDs and acetaminophen was independently associated with increased mortality.

## Abbreviations

APACHE: Acute Physiology and Chronic Health Evaluation; ICU: intensive care unit; MAX_ICU: _maximum body temperature during ICU stay; NSAIDs: non-steroidal anti-inflammatory drugs; STROBE: Strengthening the Reporting of Observational Studies in Epidemiology.

## Competing interests

All principal investigators have no financial competing interests to disclose. There are no conflicts of interest to disclose related to this investigation.

## Writing committee (contributions)

**Korea**: Younsuck Koh, M.D. PhD (Division of Pulmonary and Critical Care Medicine, Department of Internal Medicine, Asan Medical Center, University of Ulsan College of Medicine, Seoul, republic of Korea); Jae Yeol Kim, M.D. (Department of Pulmonary and Critical Care Medicine, Chung-Ang University College of Medicine, Seoul, Republic of Korea); and Gee Young Suh, M.D. (Division of Pulmonary and Critical Care Medicine, Samsung Medical Center, Sungkyunkwan University School of Medicine, Seoul, Republic of Korea).

**Japan**: Masaji Nishimura, M.D. PhD (Department of Emergency and Critical Care Medicine, Tokushima University Hospital, Tokushima, Japan) and Moritoki Egi, M.D. (Department of Intensive Care, Okayama University Hospital, Okayama, Japan).

ME and MN conceived the study. ME, MN, YK, JYK and GYS participated in the design of the study and coordinated patient enrollment and data collection for their respective countries. MN and JYK managed the data collection website for the respective countries. ME performed the statistical analyses. ME, MN, YK, JYK and GYS participated in data interpretation and drafted the manuscript. All authors read and approved the final manuscript.

## Supplementary Material

Additional file 1 **Maximum body temperature during ICU stay and 28-day mortality of patients with and without sign of infection**. Click here for file
