# Supplementary material for: Association of body temperature and antipyretic treatments with mortality of critically ill patients with and without sepsis: multi-centered prospective observational study
Source: Crit Care. 2012 Feb 28;16(1):R33. doi: 10.1186/cc11211 (PMC3396278; doi:10.1186/cc11211)
Supplement: Additional file 1 — Maximum body temperature during ICU stay and 28-day mortality of patients with and without sign of infection. [file cc11211-S1.PDF]

Maximum body temperature during ICU stay and 28-day mortality of patients with and without sign of infection.

Max. body temp. during ICU stay	Patients with suspected or proven infection (*N*=724)		Patients without sign of infection (*N*=701)	
	28-day mortality	Unadjusted odds ratio (95%CI)	28-day mortality	Unadjusted odds ratio (95%CI)
<36.5°C	2/4 (50.0%)	3.56 (0.48, 26.6) (p=0.48)	0/2 (0%)	n.a.
36.5°C –37.4°C	25/114 (21.9%)	1 (reference)	3/224 (1.3%)	1 (reference)
37.5°C–38.4°C	43/288 (14.9%)	0.62 (0.36, 1.08) (p=0.12)	14/374 (3.7%)	2.86 (0.81, 10.1) (p=0.15)
38.5°C–39.4°C	49/224 (21.9%)	1.00 (0.58, 1.72) (p=0.90)	5/86 (5.8%)	4.55 (1.06, 19.5) (p=0.003)
≥39.5°C	28/94 (29.8%)	1.51 (0.81, 2.82) (p=0.26)	2/15 (13.3%)	11.3 (1.74, 73.9) (p=0.004)

In current study, attending physician (investigators) evaluated infectious condition every day and scored as “no-sign of infection”, “suspected infection” or “culture proven infection”. This table showed that the association of maximum body temperature during ICU stay and 28-day mortality of patients with and without sign of infection.
Unadjusted odds ratio was reported relative to a reference body temperature defined as category 36.5°C–37.4°C.
ICU, intensive care unit; CI, confidential interval; n.a., not applicable (no patients in this category)
